# Evaluating the role of large language models in supporting patient education during the informed consent process for routine radiology procedures

**DOI:** 10.1093/bjr/tqaf225

**Published:** 2025-09-15

**Authors:** Eric Einspänner, Roland Schwab, Sebastian Hupfeld, Maximilian Thormann, Erelle Fuchs, Matthias Gawlitza, Jan Borggrefe, Daniel Behme

**Affiliations:** Clinic for Neuroradiology, Otto von Guericke University Magdeburg Medical Faculty, Magdeburg, 39120, Germany; Clinic for Neuroradiology, Otto von Guericke University Magdeburg Medical Faculty, Magdeburg, 39120, Germany; Department of Radiation Protection Magdeburg, University Hospital, Otto-von-Guericke-University, Magdeburg, 39120, Germany; Clinic for Neuroradiology, Otto von Guericke University Magdeburg Medical Faculty, Magdeburg, 39120, Germany; Department of Nuclear Medicine, Charité Universitaetsmedizin Berlin, Berlin, 10117, Germany; Clinic for Neuroradiology, Otto von Guericke University Magdeburg Medical Faculty, Magdeburg, 39120, Germany; Section Neuroradiology, University Hospital Jena Institute of Diagnostic and Interventional Radiology, Jena, 07747, Germany; Department of Radiology, Neuroradiology, and Nuclear Medicine, Johannes-Wesling-Hospital, Ruhr-University-Bochum, Bochum, 44789, Germany; Clinic for Neuroradiology, Otto von Guericke University Magdeburg Medical Faculty, Magdeburg, 39120, Germany; STIMULATE Research Campus, Otto von Guericke University Magdeburg, Magdeburg, 39106, Germany

**Keywords:** ChatGPT, AI, LLM, NLP, radiology

## Abstract

**Objectives:**

This study evaluated 3 LLM chatbots (GPT-3.5-turbo, GPT-4-turbo, and GPT-4o) on their effectiveness in supporting patient education by answering common patient questions for CT, MRI, and DSA informed consent, assessing their accuracy and clarity.

**Methods:**

Two radiologists formulated 90 questions categorized as general, clinical, or technical. Each LLM answered every question 5×. Radiologists then rated the responses for medical accuracy and clarity, while medical physicists assessed technical accuracy using a Likert scale. Semantic similarity was analyzed with SBERT and cosine similarity.

**Results:**

Ratings improved with newer model versions. Linear mixed-effects models revealed that GPT-4 models were rated significantly higher than GPT-3.5 (*P* < .001) by both physicians and physicists. However, physicians’ ratings for GPT-4 models showed a significant performance decrease for complex modalities like DSA and MRI (*P* < .01), a pattern not observed in physicists’ ratings. SBERT analysis revealed high internal consistency across all models. SBERT analysis revealed high internal consistency across all models.

**Conclusion:**

Variability in ratings revealed that while models effectively handled general and technical questions, they struggled with contextually complex medical inquiries requiring personalized responses and nuanced understanding. Statistical analysis confirms that while newer models are superior, their performance is modality-dependent and perceived differently by clinical and technical experts.

**Advances in knowledge:**

This study evaluates the potential of LLMs to enhance informed consent in radiology, highlighting strengths in general and technical questions while noting limitations with complex clinical inquiries, with performance varying significantly by model type and imaging modality.

## Introduction

Generative artificial intelligence (gAI) is already being used in a variety of ways to support clinical routine, such as for the preparation of findings, the summarization of disease progression, etc.^1^^,^^2^ Furthermore, gAI can be used to ensure personalized patient information for diagnostic MR, CT, and digital subtraction angiography (DSA) examinations.^3^^,^^4^ This allows detailed end-to-end conversations to be held between the patient and ChatBots in real time.

The use of gAI in the generation of patient information requires extensive and strict quality control. For example, the models are expected to generate the latest research findings and current guidelines into answers that are understandable for the patient. However, most models are not specifically designed and trained for this purpose and usually contain disclaimers that preclude their use in a clinical context.^5^ The accuracy and consistency of the answers to patient questions has already been shown in several studies.^6–8^

ChatGPT is a sophisticated language model that utilizes deep learning techniques to generate human-like responses to natural language inputs.^9^ ChatGPT belongs to the family of generative pre-training transformer (GPT) models developed by OpenAI and is one of the largest publicly available language models. Leveraging an extensive corpus of text data, ChatGPT can grasp the nuances and intricacies of human language, enabling it to generate suitable and contextually relevant responses across a wide range of prompts.^9^ ChatGPT has the potential to greatly reduce the time needed to complete various tasks. When used judiciously, the time saved by employing ChatGPT can be redirected toward more productive and higher-priority activities.^10–12^ In the context of radiology, a relevant number of working hours is spent on getting informed consent for investigations or interventions, making this process an excellent target for automation.^13^^,^^14^ Through the use of large language models (LLMs), chatbots are intended to close the information gap in patient education for various diagnostic procedures. These advanced models have the potential to provide personalized, comprehensible answers that incorporate the latest medical evidence, helping to reduce patient uncertainty.

This study seeks to evaluate how 3 publicly available LLM-based chatbots perform in responding to common questions in the context of informed consent for diagnostic CT, MRI, and DSA.

## Methods

### Collection and processing of patient questions

Common patient questions were collected from 2 different experienced radiologists for the individual modalities CT, MRI, and DSA. The aim was to formulate for each modality 15-20 of the most frequently asked patient questions based on experience, regardless of whether the questions were medical, technical-physical, or general. Duplicate and similarly worded questions were then removed by a third radiologist with 8 years of professional experience. This resulted in 29 questions for CT, 30 questions for MRI, and 31 questions for DSA, which were formulated in German and English, respectively (see Table 1). The English translations of the questions were reviewed by a native English speaker. For a more detailed analysis of the usability of the individual models, the questions were divided into further categories: general, hospital-specific and physical questions (Table S1). The 3 common models GPT3.5-turbo, GPT4-turbo, and GPT4o from OpenAI^15^ were then compared to each other. Model responses were generated via the OpenAI API, which provides programmatic access to models through HTTPS requests. Prompts were submitted as text strings using Python, and the API returned generated outputs as JSON-formatted responses. No local model hosting or fine-tuning was performed; all model inference occurred on OpenAI's hosted infrastructure.

**Table 1.** Extract from the respective question catalogue.

CT	MRI	DSA
What is the risk of radiation during a CT scan?	Is it cramped in the MRI scanner?	What exactly does DSA mean?
What is the risk of a contrast agent allergy?	How loud is it in the MRI scanner?	Is the contrast agent dangerous for my kidneys?
What is the risk of having a reaction to contrast agents?	I have a pacemaker, can I have an MRI scan?	Will I notice any side effects from the DSA examination?
I am pregnant, can I still have a CT scan?	I have an implanted defibrillator, can I have an MRI?	Is the DSA examination painful?

Each of the 5 repeated queries per question was submitted as a separate, independent API call. We queried the models with all parameters left at their default values. No random seed was set for repeat calls, and each question was submitted once per model. As a result, responses reflect the stochastic nature of autoregressive text generation under typical usage conditions. Since the API is stateless and does not retain contextual memory between requests, no specific time interval between calls was required or controlled. In order to adapt the answers as precisely as possible to our requirements, background information was formulated and sent together with the respective question. The exact formulation of the context based question is shown in Table 2.

**Table 2.** Context formulation in the respective language.

German	English
Du bist Jarvis, ein Radiologie Facharzt.Deine Aufgabe ist es, die Fragen der Patienten in einem Aufklärungsgespräch zur {modality} {method} zu beantworten. Deine Antworten sollten verständlich und empathisch sein. Patient: {question}Jarvis:	You are Jarvis, a radiology specialist. Your job is to answer patients’ questions in an information session about {modality} {method}.Your answers should be understandable and empathetic. Patient: {question}Jarvis:

The placeholder modality, method and question are replaced according to the question.

For example, this results in a question from the field of diagnostic DSA:

*“You are Jarvis, a radiology specialist. Your job is to answer patients’ questions in an information session about DSA diagnostics. Your answers should be understandable and empathetic.*

*Patient: What exactly does DSA mean?*

*Jarvis: {response}”*

The question and the answer were then saved accordingly in a log file.

### Rating of responses

- The 5 answers to the individual questions were assessed by 2 experienced radiologists in terms of medical accuracy and clarity using the following Likert scale: “excellent response not requiring clarification”
- “very good response requiring very minimal clarification”
- “satisfactory requiring minimal clarification”
- “partially satisfactory requiring moderate clarification”
- “unsatisfactory requiring substantial clarification”

For this purpose we categorized the responses similarly to Mika et al^16^ and Lang et al.^17^ In addition, the technical and radiation protection issues were also assessed by 2 medical physics experts with experience in X-ray and MRI.^18^ Inter-rater reliability was calculated using Python and the Pingouin module.

A comparison of the answers was then carried out. SBERT, a framework for computing sentence embeddings using the BERT model, was used for this purpose. In this study, we utilized the SentenceTransformer model to compute semantic similarities between pairs of sentences. The specific model employed was paraphrase-MiniLM-L6-v2,^19^ which was designed for generating sentence embeddings that capture the semantic meaning of sentences.^20^ To assess the semantic similarity between the sentences, we calculated the cosine similarity using PyTorch. Cosine similarity is a metric that measures the cosine of the angle between 2 non-zero vectors, providing a value between −1 and 1, where 1 indicates identical meaning, 0 indicates no similarity, and −1 indicates opposite meanings.^21^

Finally, the answers were compared with each other. The reason for the comparison is to determine how much the response quality of the individual models varies. Usually, you only ask the question once to the chatbot and expect the best possible answer, without a strong variance in terms of accuracy and clarity. The mean and the standard deviation were then calculated from the resulting values.

### Rating of responses

To address potential interdependencies in the data, linear mixed-effects models were used to analyze ratings, stratified by rater group (physicians *vs*. physicists). The models included fixed effects for the LLM Model (GPT-3.5-turbo, GPT-4-turbo, GPT-4o), Modality (CT, MRI, DSA), and their interaction term (Model × Modality). To account for repeated measurements, Question was included as a random intercept.

To formally test for differences in response consistency, we conducted a 1-way analysis of variance (ANOVA) to compare the mean semantic similarity scores across the 3 models.

## Results

### Rating

**Figure 1.**
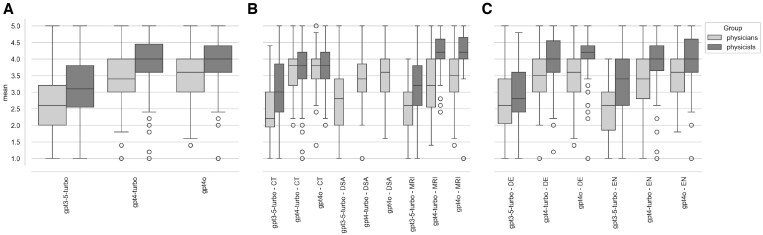
Mean rating scores of all raters. The red boxes represent the ratings provided by physicians, while the blue boxes correspond to the ratings from physicists. The 3 panels present the mean rating by model (A), model-modality (B), and model-language (C).

We conducted a comparison of 3 different LLM models; GPT3.5-turbo, GPT4-turbo, and GPT4o, evaluating their performance across 3 imaging modalities; CT, DSA, and MRI. Two different groups made up of physicists and physicians, were asked to rate the models based on their respective expertise. The physicists primarily assessed the models’ performance on questions related to radiation protection, radiation dose, and technical aspects, while physicians focused on evaluating the medical relevance of the answers. The boxplots in Figure 1 illustrate the comparison of mean scores between these 2 groups. For physicians, the Intraclass Correlation Coefficient (ICC1k, average raters absolute) was 0.66, indicating moderate agreement. For physicists, the ICC was 0.83, indicating good agreement.

Across all models (GPT3.5-turbo, GPT4-turbo, and GPT4o), physicists rated responses to technical and radiation-related questions higher than the physicians rated medical-related questions (see Figure 1A). The variability in the scores is also evident, with wider interquartile ranges for the physicians in GPT4-turbo and GPT4o compared to the physicists.

To formally test these observations, the ratings were analyzed using linear mixed-effects models, stratified by rater group. The analysis of the physicians’ ratings confirmed that the newer GPT-4 models were rated significantly higher than GPT-3.5, with beta values of 1.225 for GPT-4-turbo (95% CI, 0.965-1.485, *P < .*001) and 1.275 for GPT-4o (95% CI, 1.014-1.535, *P < .*001). However, the performance of these advanced models was modality-dependent, showing significantly reduced scores for complex modalities like DSA. The negative interaction for GPT-4o and DSA (β = −0.486, 95% CI, −0.793 to −0.179, *P* = .002) indicates that the models’ effectiveness diminishes when faced with more specialized clinical questions.

For the physicists’ ratings, a similar pattern of overall improvement was observed, with GPT-4 models also scoring significantly higher than GPT-3.5 (GPT-4-turbo: β = .548, 95% CI, 0.332-0.764, *P < .*001; GPT-4o: β = .738, 95% CI, 0.523-0.953, *P < .*001). In contrast to the physicians’ assessment, GPT-4-turbo showed a significantly *enhanced* performance for MRI-related tasks (β = .505, 95% CI, 0.195-0.814, *P = .*001), while the improvement for GPT-4o on MRI questions was not statistically significant (*P = .*149). These findings confirm that while newer models are generally superior, their performance is perceived differently by clinical and technical experts, particularly in relation to specific imaging modalities.

Across all results, physicists provide higher ratings on average compared to physicians, with noticeable variations in the different models. Outliers are more prevalent in the physicians’ ratings, indicating occasional extreme values, while physicists’ ratings show greater consistency.

Table 3 shows that the answers from GPT3.5-turbo were rated the lowest for all 3 categories. The quality of the answers improved with each model generation, with GPT-4o achieving the highest rating. Using GPT-4o 77.8% of general questions were rated with a median score of 4 or higher.

**Table 3.** Proportion of questions with a median of ≥4 across all 5 answers in the corresponding total pool.

	GPT3.5-turbo	GPT4-turbo	GPT4o
General	35.8%	63.9%	77.8%
Medical	17%	39.9%	53.4%
Physics	22.7%	48.7%	65.7%

### SBERT

**Figure 2.**
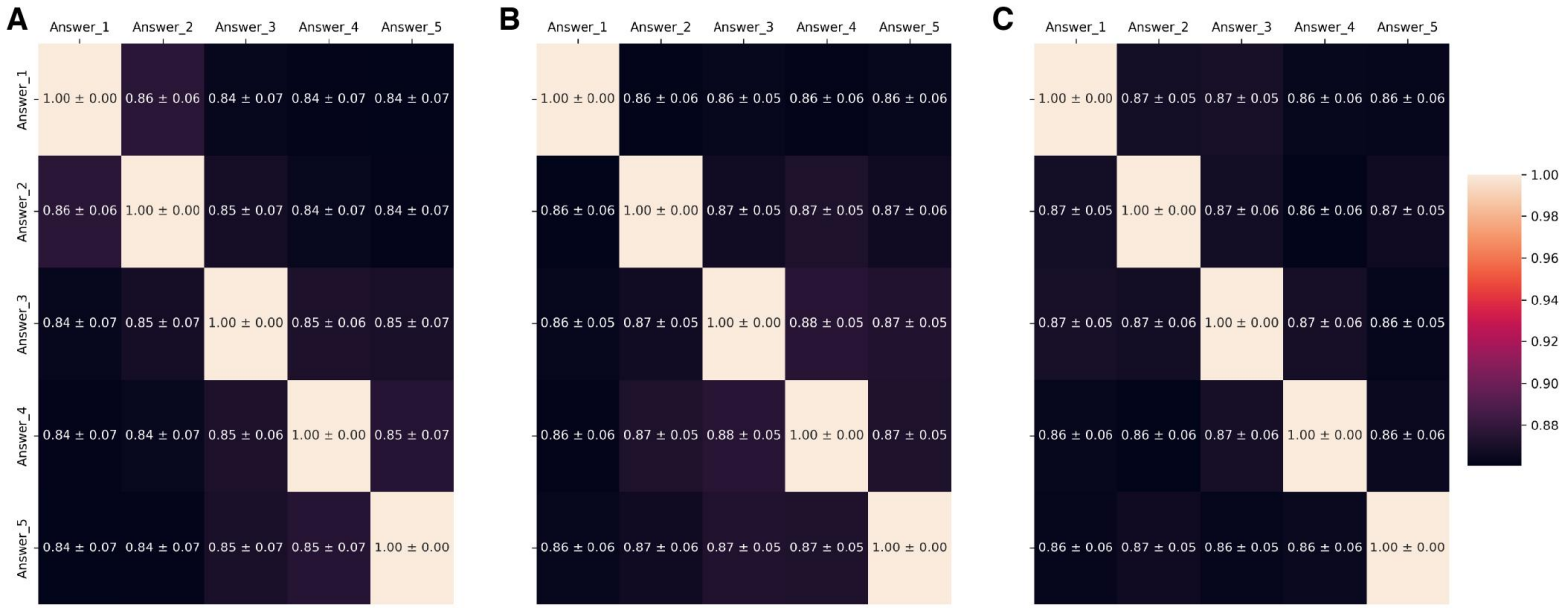
Similarity scores (mean ± std dev) for GPT3.5-turbo (A), GPT4-turbo (B), and GPT4o (C).

The heat maps of the pairwise similarity comparisons within the models show that all models are fairly consistent (see Figure 2). As expected, the diagonals show maximum similarities (1.00), while the pairwise comparisons show similarity values between 0.84 and 0.88, which indicates a high internal consistency of the generated responses. This also shows that GPT4o has slightly higher mean similarities than GPT4-turbo and GPT3.5-turbo.

**Figure 3.**
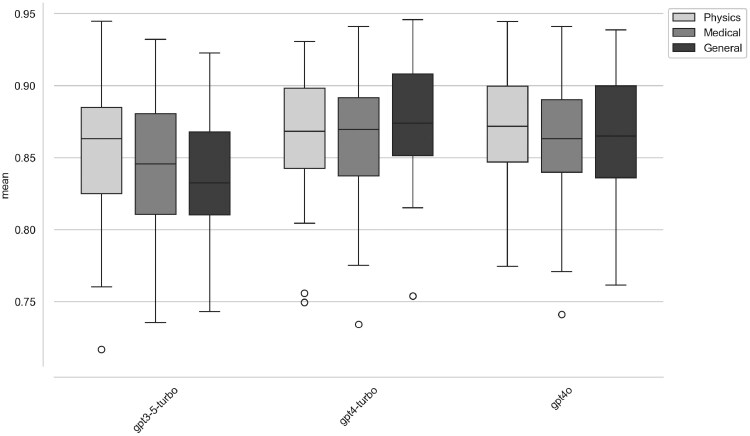
Similarity scores for all 3 models and question groups.

The results of the similarity analysis of the 3 GPT models (GPT3.5-turbo, GPT4-turbo, and GPT4o) based on SBERT and cosine similarity show consistently high similarity values. All similarity values were between 0.55 (min) and 0.98 (max). This is reflected in the boxplots (see Figure 3), which show the mean values of the similarities per model for the different content groups (physics, medicine, general). The distribution of similarities remains within the range of 0.80 to 0.95, with values below 0.80 occurring in a few cases, as can be seen from the outliers in the boxplots. To formally test for differences in response consistency, an ANOVA was conducted on the similarity scores. The test revealed a statistically significant difference between the models (*F* = 82.13, *P < .*0001). A subsequent Tukey’s HSD *post hoc* test showed that both GPT-4-turbo and GPT-4o produced significantly more consistent responses than GPT-3.5-turbo (*P < .*001 for both comparisons). However, the difference in consistency between GPT-4-turbo and GPT-4o was not statistically significant (*P = .*2026).

## Discussion

ChatGPT offers both opportunities and challenges for clinical patient care. For example, tasks in radiology could become automated with sufficient quality. Some of ChatGPT’s skills include creating reports, extracting relevant information from reports, as well as providing decision support and answering patient questions.^22–24^ However, none of these studies focused on ChatGPT’s ability to obtain informed consent in a radiology setting. This is a significant aspect of a radiologist’s daily routine.

This study is one of the first to demonstrate the ability of ChatGPT to answer commonly asked questions in the context of obtaining informed consent for routine radiologic examinations. Our results emphasize the versatility of ChatGPT in the clinical setting. Our results also show that the latest versions, GPT4-turbo and GPT4o, show significant improvement compared to GPT3.5-turbo in all question categories, especially for general questions. There are already a few studies^25^^,^^26^ that focus on similar topics, but none specific to general diagnostic radiology patient education.

### Rating

In general, the proportion of very good and excellent answers in all 3 subclasses increases with increasing version number. This shows the further development of the LLM with a growing database and continuous optimization of the algorithm. General questions have the highest proportion of very good or excellent answers across all 3 models (35.8%-77.8%). This is closely followed by the physics questions (22.7%-65.7%). The specific medical questions have the lowest frequency of good answers being given (17%-53.4%). The improved performance of GPT4 compared to GPT3.5 has also been shown in other studies.^24^^,^^27–29^ For example, Krishna et al^29^ shows that GPT4 delivers more consistent responses than GPT3.5 across 3 trials. This is an observation that can also be made from our study, in particular to the increased performance from GPT4-turbo to GPT4o.

Our statistical analysis provides robust evidence for this trend, confirming that GPT-4-turbo and GPT-4o significantly outperformed GPT-3.5 from the perspective of both physicians and physicists. However, the linear mixed-effects models reveal a more complex picture. The significant negative interaction between advanced models and complex modalities (like DSA) in the physicians’ ratings suggests that while the models have improved, their ability to handle nuanced, high-stakes clinical questions remains limited. Surprisingly, the physicists’ ratings did not show the same performance drop for complex modalities. In fact, GPT-4-turbo’s performance was significantly better for technical MRI questions. This divergence highlights a key finding: the models’ effectiveness is highly context-dependent and is judged differently based on professional expertise level.

General questions can be answered well without requiring specific specialized knowledge or and do patient’s specific context situation. For example, questions about the volume of the modalities or the size of the scanner can be based on manufacturer information without the need for contextualization. A similar point applies to most physical questions. There are fixed thresholds or guidelines and the models, particularly GPT4o and GPT4-turbo, that perform well in answering technical and radiation-related questions. This aligns with physicists expertise and expectations. In Laukamp et al,^30^ where the ability of GPT4 to produce comprehensive summaries of patient histories was evaluated from multiple MRI reports, it turns out that ChatGPT can achieve more satisfactory results when given available information (context). Comparing this to our results, it is noticeable that the generated output without additional information input, just based on questions, does not match the quality of the results from Laukamp et al.^30^

The physicians consistently rated the models lower for specific medical questions. This could indicate that while the models are proficient at answering questions related to physics and technical considerations, there is room for improvement in how they handle clinically relevant questions. For example, if a patient asks whether their known renal impairment is relevant to an examination requiring the administration of contrast media, individual factors such as glomerular filtration rate, long-term medication and other pre-existing diseases need to be weighed in to provide an adequate answer. Only then can the answer be adequately made in the context of the specific examination. Such questions are more complex and require deeper context or understanding. This is also confirmed by Cozzi et al.^28^ Although they found an improvement in reasoning skills with newer tasks, they also recognized difficulties with more complex clinical tasks. Physicians rated GPT4o the highest among the 3 models, however, the variability in their responses suggests that some of the answers did not fully meet their clinical needs. Our use of a structured system prompt was designed to emulate a realistic clinical context where a healthcare professional is aware of key information (eg, imaging modality) before a conversation begins. This approach ensures a controlled, replicable evaluation aligned with the intended deployment scenario, rather than introducing artificial performance inflation. While an open-ended, patient-driven dialogue represents a different use case, our study focuses on a standardized informative interaction appropriate for the use in clinical education.

### SBERT

The semantic similarity of responses is a direct measure of the consistency and reliability of the information provided by the LLM. For consent to be considered legally and ethically valid, it must be informed. High semantic similarity across multiple answers to the same query ensures that the user receives a stable and unambiguous message, reducing the risk of confusion and allowing for clear comprehension. Conversely, low semantic similarity would imply that the model provides contradictory or inconsistent information, which could mislead the user and fundamentally undermine their ability to make a truly informed decision. Therefore, evaluating this metric is crucial for assessing the system’s fitness for generating information related to user consent.

The pairwise comparisons using SBERT and cosine similarity make it clear that the models are able to generate similar response patterns. This speaks in favor of a robust performance, regardless of the specific model version. GPT4o tends to show slightly better consistency compared to the other models, which could be of interest in a clinical context. The results showed that in a clinical context, where only 1 answer is usually given to the corresponding question, the first answer does not differ semantically from the others.

One limitation of this analysis is the consideration of mean values, which cannot reflect all the subtleties in the variability of the responses.

## Conclusion

In conclusion, while the newer models (GPT4o and GPT4-turbo) offer improvements in both technical and medical domains, there remains a gap between their performance on technical *versus* clinical questions. For general questions, however, the results are promising and could be used in everyday clinical practice. The data suggest that generative models can be highly useful in supporting technical assessments, particularly in fields like radiation protection or technical aspects. However, further refinement is needed for medical applications, where more complex, context-sensitive information is required. This indicates the importance of continuing to enhance these models for clinical relevance, while acknowledging their strengths in technical and radiological areas.

### Ethical and regulatory considerations

The utilization of LLMs in patient education requires careful consideration of ethical and regulatory frameworks. A critical aspect is medicolegal liability; healthcare professionals remain responsible for verifying and contextualizing AI-generated information. Unvetted outputs could lead to patient harm and legal ramifications. Furthermore, data protection compliance, particularly with regulations like GDPR, is essential when these tools process personal health data. Finally, any AI-generated content must align with established consent guidance, such as that from the UK's General Medical Council (GMC). This means the AI must support—not replace—the personalized, iterative process of shared decision-making, which is the cornerstone of valid informed consent. Safe utilization therefore requires robust frameworks incorporating clinician oversight, patient safeguards, and continuous validation.

### Limitations

In our study, we only evaluated the responses to individual questions and did not simulate a real dialogue, starting with an informational conversation followed by patient questions. The question set was generated by radiologists, not derived from a patient corpus, which limits the external validity and may not fully capture the spectrum of patient concerns or health literacy levels. Additionally, the answer quality of the models is highly dependent on the created context. Furthermore, these are also models without specific fine tuning. These 2 limitations, context and fine tuning, are also emphasized in the works of Bhayana^22^ and Fink et al.^24^ We internally experimented with various context formulations and ultimately chose the one used here. We did not perform systematic optimization.

We have not checked any time dependencies of the responses with regard to their quality. It can be assumed that the latest models, which we used via API, will be further changed by OpenAI through new builds, which in turn may affect the response quality. However, it is possible to use specific models (builds) directly via API, eg, gpt-4-turbo-2024-04-09. These should not change further over time and would be best suited for use in everyday clinical practice without constant reassessment.

Due to the nature of the data collection and the subsequent categorization into subgroups, the groups were not balanced among each other. The rating was carried out by native German speakers with at least C1-level English proficiency, but no native English speakers, which may introduce subtle bias in the evaluation of English-language tone and phrasing.

### Outlook

So far, only the accuracy and clarity of individual models have been evaluated, but the direct interaction with patients has not been investigated. In the future, we want to use an experimental setup to have entire educational conversations conducted by language models, and then evaluate them.

In addition to the diagnostic questions, we also want to investigate patient questions in connection with interventional procedures. Furthermore, we would like to make a comparison with LLMs specializing in medicine, eg, Med-Gemini,^31^ which was not yet publicly available at the time of our evaluation.

## Supplementary Material

tqaf225_Supplementary_Data
