# Supplementary material for: Evaluating the role of large language models in supporting patient education during the informed consent process for routine radiology procedures
Source: Br J Radiol. 2025 Sep 15;98(1176):2184–90. doi: 10.1093/bjr/tqaf225 (PMC12810871; doi:10.1093/bjr/tqaf225)
Supplement: tqaf225_Supplementary_Data [file tqaf225_supplementary_data.docx]

| **CT** | |
| --- | --- |
| German | English |
| general | |
| Tut eine CT Untersuchung weh? | Does a CT scan hurt? |
| Wie lange dauert eine CT Untersuchung? | How long does a CT scan take? |
| Ist es eng im CT Gerät? | Is it cramped in the CT scanner? |
| Ist die CT Röhre geschlossen? | Is the CT tube closed? |
| Hilft es wenn ich die Augen während der CT Untersuchung schließe? | Does it help if I close my eyes during the CT scan? |
| Ich habe Platzangst, macht das Probleme bei der CT Untersuchung? | I have claustrophobia, will this cause problems during the CT scan? |
| clinic | |
| Wie hoch ist das Risiko einer Kontrastmittel Allergie? | What is the risk of a contrast agent allergy? |
| Wie hoch ist das Risiko auf Kontrastmittel zu reagieren? | What is the risk of having a reaction to contrast agents? |
| Kann ich nach einer CT Untersuchung noch Autofahren? | Can I still drive after a CT scan? |
| Ich habe eine Kontrastmittel Allergie, kann man die Untersuchung trotzdem mit Kontrastmittel durchführen? | I have an allergy to contrast agents, can the examination still be carried out with contrast agents? |
| Die Untersuchung ist für mein Kind, wie hoch ist die Wahrscheinlichkeit, dass mein Kind Krebs bekommt wegen der CT Untersuchung? | The examination is for my child, how high is the probability that my child will get cancer because of the CT examination? |
| Bei einer anderen Untersuchung ist mir durch Kontrastmittel schlecht geworden, ist das eine Allergie? | During another examination, the contrast agend made me sick, is this an allergy? |
| Reicht eine Röntgenuntersuchung für die Nasennebenhöhlen nicht aus oder muss es ein CT sein? | Is an X-ray examination not sufficient for the paranasal sinuses or does it have to be a CT? |
| physic | |
| Wie hoch ist das Risiko durch die Bestrahlung bei einem CT? | What is the risk of radiation during a CT scan? |
| Ich bin schwanger, kann man trotzdem ein CT machen? | I am pregnant, can I still have a CT scan? |
| Ich habe ein Tatoo, ist das ein Problem bei einer CT Untersuchung? | I have a tattoo, is that a problem during a CT scan? |
| Ich habe einen Herzschrittmacher, ist das ein Problem bei einer CT Untersuchung? | I have a pacemaker, is that a problem during a CT scan? |
| Ich habe eine Bonebridge, ist das ein Problem bei einer CT Untersuchung? | I have a bonebridge, is that a problem during a CT scan? |
| Ich habe ein Cochleaimplantat, ist das ein Problem bei einer CT Untersuchung? | I have a cochlear implant, is that a problem during a CT scan? |
| Ich habe Piercings, ist das ein Problem bei einer CT Untersuchung? | I have piercings, is that a problem during a CT scan? |
| Ich fliege regelmäßig, ist meine kumulative Dosis mit dem CT zusammen gefährlich? | I fly regularly, is my cumulative dose together with the CT scan dangerous? |
| Brauche ich bei einer CT Untersuchung einen Strahlenschutz für meine Hoden? | Do I need radiation protection for my testicles during a CT scan? |
| Brauche ich bei einer CT Untersuchung einen Strahlenschutz für meine Schildrüse? | Do I need radiation protection for my thyroid gland during a CT scan? |
| Brauche ich bei einer CT Untersuchung einen Strahlenschutz für meine Ovarien? | Do I need radiation protection for my ovaries during a CT scan? |
| Kann ich als Schwangere ein CT des Kopfes machen, oder ist das gefährlich? | Can I have a CT scan of my head as a pregnant woman, or is it dangerous? |
| Ich habe schon 20 CT Untersuchungen gehabt dieses Jahr, ist das ein Problem hinsichtlich der kumulativen Dosis? | I have already had 20 CT scans this year, is this a problem in terms of the cumulative dose? |
| Wie hoch ist die Dosis für ein Schädel CT ? | How high is the radiation dose for a cranial CT? |
| Wie hoch ist die Dosis für ein CT der Nasennebenhöhlen? | How high is the radiation dose for a CT of the paranasal sinuses? |
| Ist die Dosis im CT höher als im Röntgen? | Is the radiation dose in a CT scan higher than in an X-ray? |
| **DSA** | |
| German | English |
| general | |
| Was genau bedeutet DSA? | What exactly does DSA mean? |
| Merke ich etwas von der DSA Untersuchung? | Will I notice any side effects from the DSA examination? |
| Ist die DSA Untersuchung schmerzhaft? | Is the DSA examination painful? |
| Wie lange dauert die DSA Untersuchung? | How long does the DSA examination take? |
| Ist die DSA Untersuchung gefährlich? | Is the DSA examination dangerous? |
| Darf ich vor der DSA Untersuchung etwas essen? | Can I eat before the DSA examination? |
| Darf ich vor der DSA Untersuchung etwas trinken? | Can I drink before the DSA examination? |
| Bin ich während der DSA Untersuchung wach? | Will I be awake during the DSA examination? |
| Kann ich mich während der DSA Untersuchung bewegen? | Can I move during the DSA examination? |
| Ist es eng in einem DSA Gerät? | Is it cramped in a DSA machine? |
| clinic | |
| Ist das Kontrastmittel gefährlich für meine Nieren? | Is the contrast agent dangerous for my kidneys? |
| Was kann im schlimmsten Fall während einer DSA passieren? | What is the worst that can happen during a DSA? |
| Was ist ein Schlaganfall? | What is a stroke? |
| Was passiert, wenn ein Thrombus ein Gefäß verschließt? | What happens if a thrombus blocks a vessel? |
| Wie hoch ist das Risiko einer Komplikation während der DSA? | How high is the risk of complications during a DSA? |
| Welche Risiken gibt es bei einer DSA? | What are the risks of a DSA? |
| Kann ich meine Medikamente vor und nach der DSA Untersuchung weiternehmen? | Can I continue to take my medication before and after the DSA examination? |
| Was kann ich nach der DSA Untersuchung machen? | What can I do after the DSA test? |
| Kann ich nach der DSA Untersuchung aufstehen oder sitzen? | Can I stand up or sit down after the DSA examination? |
| Kann ich nach der DSA Untersuchung allein auf die Toilette gehen? | Can I go to the toilet on my own after the DSA examination? |
| Wie lange muss der Druckverband anliegen? | How long does the pressure bandage have to be in place? |
| Wann kann ich nach der DSA Untersuchung nachhause? | When can I go home after the DSA examination? |
| Wie häufig werden DSA Untersuchungen durchgeführt? | How often are DSA examinations carried out? |
| Wie läuft eine diagnostische DSA ab? | How does a diagnostic DSA work? |
| Wie groß sind die Katheter bei einer DSA Untersuchung? | How large are the catheters during a DSA examination? |
| Ist das Kontrastmittel bei einer DSA schädlich? | Is the contrast agent harmful during a DSA? |
| Muss ich meine blutverdünnende Medikamente bei einer DSA Untersuchung absetzen? | Do I have to stop taking my blood-thinning medication during a DSA examination? |
| Warum reicht ein MRT nicht aus, muss es eine DSA Untersuchung sein? | Why is an MRI not enough, does it have to be a DSA examination? |
| Kann man nicht ein CT anstatt einer DSA machen? | Is it not possible to do a CT instead of a DSA? |
| physic | |
| Wie groß ist die Strahlenbelastung bei einer DSA Untersuchung? | How high is the radiation exposure during a DSA examination? |
| Sind die Röntgenstrahlen gefährlich? | Are X-rays dangerous? |
| **MRI** | |
| German | English |
| general | |
| Wie eng ist es im MRT? | Is it cramped in the MRI scanner? |
| Wie laut ist es im MRT? | How loud is it in the MRI scanner? |
| Ich habe Platzangst, gibt es da etwas, was mir helfen kann, die Untersuchung trotzdem auszuhalten? | I have claustrophobia, is there anything that can help me to endure the examination anyway? |
| Was passiert, wenn ich die Enge im Gerät nicht aushalte? | What happens if I can't stand the tightness in the device? |
| Muss ich den Gürtel meiner Hose auch ausziehen? | Do I also have to take off the belt of my trousers? |
| Sind die Knöpfe an der Hose ein Problem für das MRT? | Are the buttons on my trousers a problem for the MRI? |
| Kann ich Musik hören während der Untersuchung? | Can I listen to music during the examination? |
| Kann ich meine Beine bewegen während der MRT Kopfuntersuchung? | Can I move my legs during the MRI head examination? |
| clinic | |
| Ich bin schwanger in der 6ten Woche, ist das ein Problem für das MRT? | I am 6 weeks pregnant, is that a problem for the MRI? |
| Ich bin schwanger in der 20ten Woche, ist das ein Problem für das MRT? | I am 20 weeks pregnant, is this a problem for the MRI? |
| Ich habe gehört, dass es bei der mehrfachen Gabe von Gadolinium zu Ablagerungen im Gehirn kommt, ist das gefährlich? | I have heard that the multiple administration of gadolinium causes deposits in the brain, is that dangerous? |
| Ich habe eine Allergie gegen jodhaltiges Kontrastmittel, ist das ein Problem im MRT? | I have an allergy to iodine-containing contrast agents, is this a problem in the MRI? |
| Meine glomeruläre Filtrationsrate ist 35, kann ich Kontrastmittel bekommen? | My glomerular filtration rate is 35, can I have contrast agents? |
| Meine glomeruläre Filtrationsrate ist 15, kann ich Kontrastmittel bekommen? | My glomerular filtration rate is 15, can I have contrast agents? |
| Meine glomeruläre Filtrationsrate ist 5, kann ich Kontrastmittel bekommen? | My glomerular filtration rate is 5, can I get contrast agents? |
| Ich habe eine MS und brauche eine Verlaufskontrolle, brauche ich unbedingt immer Untersuchungen mit Kontrastmittel? | I have MS and need a follow-up, do I always need to have contrast agents? |
| Sind die elektromagnetischen Wellen krebserregend? | Are the electromagnetic waves carcinogenic? |
| physic | |
| Ich habe einen Herzschrittmacher, darf ich in ein MRT? | I have a pacemaker, can I have an MRI scan? |
| Ich haben einen implantierten Defibbrilator, darf ich in das MRT? | I have an implanted defibbrilator, can I have an MRI? |
| Ich habe ein Tatoo, ist das ein Problem für das MRT? | I have a tattoo, is that a problem for the MRI? |
| Ich habe ein Piercing, ist das ein Problem für das MRT? | I have a piercing, is that a problem for the MRI? |
| Ich habe eine Zahnspange, ist das ein Problem für das MRT? | I have braces, is that a problem for the MRI? |
| Ich habe Granatsplitter in der Haut, ist das ein Problem für das MRT? | I have shrapnel in my skin, is that a problem for the MRI? |
| Ich habe eine antikontrazeptive Spirale, ist das ein Problem fürs MRT? | I have an anticontraceptive coil, is that a problem for the MRI? |
| Ich habe eine spinale Schmerzpumpe, ist das ein Problem fürs MRT? | I have a spinal pain pump, is that a problem for the MRI? |
| Ich habe Hüftimplantate aus dem Jahr 1960, ist das ein Problem für das MRT? | I have hip implants from 1960, is this a problem for the MRI? |
| Ich habe Hüftimplantate aus dem Jahr 2020, ist das ein Problem für das MRT? | I have hip implants from 2020, is that a problem for the MRI? |
| Darf ich meine Sauerstoffflasche mit ins MRT nehmen? | Can I take my oxygen bottle with me into the MRI? |
| Ich bin auf einen Rollstuhl angewiesen, kann ich damit bis an die Liege des MRTs gefahren werden? | I am dependent on a wheelchair, can I be driven right up to the MRI couch? |
| Wird es warm während der MRT Untersuchung? | Does it get warm during the MRI scan? |

**Table S1:** Frequently asked patient questions for CT, MRI and DSA examinations, categorized into general, clinic and physic.
